# A Recombinant Positive Control for Serology Diagnostic Tests Supporting Elimination of *Onchocerca volvulus*

**DOI:** 10.1371/journal.pntd.0004292

**Published:** 2016-01-08

**Authors:** Allison Golden, Eric J. Stevens, Lindsay Yokobe, Dunia Faulx, Michael Kalnoky, Roger Peck, Melissa Valdez, Cathy Steel, Potochoziou Karabou, Méba Banla, Peter T. Soboslay, Kangi Adade, Afework H. Tekle, Vitaliano A. Cama, Peter U. Fischer, Thomas B. Nutman, Thomas R. Unnasch, Tala de los Santos, Gonzalo J. Domingo

**Affiliations:** 1 Diagnostics Global Program, PATH, Seattle, Washington, United States of America; 2 Laboratory of Parasitic Diseases, National Institute of Allergy and Infectious Diseases, National Institutes of Health, Bethesda, Maryland, United States of America; 3 National Onchocerciasis Control Programme, Kara, Togo; 4 Onchocerciasis Reference Laboratory, National Institute of Hygiene, Sokodé, Togo; 5 Institute of Tropical Medicine, University Clinics of Tübingen, Tübingen, Germany; 6 African Programme for Onchocerciasis Control, World Health Organization, Ouagadougou, Burkina Faso; 7 Division of Parasitic Diseases and Malaria, Centers for Disease Control and Prevention, Atlanta, Georgia, United States of America; 8 Department of Internal Medicine, Infectious Diseases Division, Washington University School of Medicine, St. Louis, Missouri, United States of America; 9 Global Health Infectious Disease Research Program, Department of Global Health, University of South Florida, Tampa, Florida, United States of America; Michigan State University, UNITED STATES

## Abstract

**Background:**

Serological assays for human IgG4 to the *Onchocerca volvulus* antigen Ov16 have been used to confirm elimination of onchocerciasis in much of the Americas and parts of Africa. A standardized source of positive control antibody (human anti-Ov16 IgG4) will ensure the quality of surveillance data using these tests.

**Methodology/Principal Findings:**

A recombinant human IgG4 antibody to Ov16 was identified by screening against a synthetic human Fab phage display library and converted into human IgG4. This antibody was developed into different positive control formulations for enzyme-linked immunosorbent assay (ELISA) and rapid diagnostic test (RDT) platforms. Variation in ELISA results and utility as a positive control of the antibody were assessed from multiple laboratories. Temperature and humidity conditions were collected across seven surveillance activities from 2011–2014 to inform stability requirements for RDTs and positive controls. The feasibility of the dried positive control for RDT was evaluated during onchocerciasis surveillance activity in Togo, in 2014. When the anti-Ov16 IgG4 antibody was used as a standard dilution in horseradish peroxidase (HRP) and alkaline phosphatase (AP) ELISAs, the detection limits were approximately 1ng/mL by HRP ELISA and 10ng/mL by AP ELISA. Positive control dilutions and spiked dried blood spots (DBS) produced similar ELISA results. Used as a simple plate normalization control, the positive control antibody may improve ELISA data comparison in the context of inter-laboratory variation. The aggregate temperature and humidity monitor data informed temperature parameters under which the dried positive control was tested and are applicable inputs for testing of diagnostics tools intended for sub-Saharan Africa. As a packaged positive control for Ov16 RDTs, stability of the antibody was demonstrated for over six months at relevant temperatures in the laboratory and for over 15 weeks under field conditions.

**Conclusions:**

The recombinant human anti-Ov16 IgG4 antibody-based positive control will benefit inter-laboratory validation of ELISA assays and serve as quality control (QC) reagents for Ov16 RDTs at different points of the supply chain from manufacturer to field use.

## Author Summary

Serological markers such as antibody responses to pathogen-specific antigens are used to inform disease epidemiology in many elimination programs. A major challenge with program-scale serological testing, and with any diagnostic test validation, is access to consistent and unlimited control reagents with which to provide assay QC and facilitate data consolidation. In the context of disease elimination, clinical positive sera will be particularly difficult to source and use as routine, inter-laboratory reagents. This study reports on a recombinant antibody specific against a key serological marker for onchocerciasis: its selection, testing, and incorporation into protocols across relevant immunoassay platforms. We have demonstrated it is a viable reagent for integration into QC and QA protocols to support long-term serological testing for onchocerciasis to support disease elimination efforts. This approach should be generalizable to other diagnostic tools supporting programs to achieve the 2020 goals of the London Declaration on Neglected Tropical Diseases.

## Introduction

Onchocerciasis, or “river blindness,” is a disease caused by the filarial parasite *O*. *volvulus* (Ov) that affects an estimated 37 million (2005) in Africa and a few thousand people in the Americas and Yemen [1,2]. In recent years, the burden of disease has been reduced significantly through large programs of community-directed treatment with ivermectin (CDTI), an antiparasitic drug donated by Merck. Data from both the Americas and Africa suggest that elimination may be achieved in the most part through mass drug administration (MDA) [3–9]. However, in some scenarios, such as where there is *Loa loa* co-endemicity, other interventions may be required.

Active infection is detected by direct observation of the Ov microfilariae (MF) through skin snip combined with microscopy. This method is not very sensitive, especially when microfilarial (MF) skin densities are low, which are typical in low-transmission settings. Polymerase chain reaction-based assays of skin snips can significantly increase sensitivity [10,11] but are not suitable for either surveillance or point of care. Consequently, serological assays have been adopted by onchocerciasis elimination programs such as the Onchocerciasis Elimination Program in the Americas (OEPA) to inform whether elimination has been achieved. Specifically, IgG antibodies to the *O*. *volvulus* antigen Ov16 are used as a marker for exposure to infection, when applied to a sentinel population of children under ten years of age as a marker of continued transmission [12,13]. Currently this test is performed as an enzyme immunoassay (EIA or ELISA plate format), both in the Americas and more recently in Africa [4,5,14,15]. It was previously demonstrated that this same assay could be transferred to the lateral flow platform to generate an RDT for anti-Ov16 IgG4 antibody [16–18]. More recently, PATH and Standard Diagnostics (Yongin-si, Gyeonggi-do, South Korea) announced the commercial availability of an Ov16 RDT. This test, called the SD BIOLINE Onchocerciasis IgG4 test, detects anti-Ov16 IgG4 in a finger-prick blood sample.

A major challenge in the standardization and inter-assay comparison of serological tests such as ELISA is the lack of a standardized positive control [19,20]. Positive controls are typically made by pooling sera from exposed individuals and are distributed either as part of a commercial kit or from a reference laboratory. While this approach is common practice, it requires access to clinical specimens, something that will get increasingly harder as onchocerciasis prevalence continues to decline. Furthermore, each new lot of positive control from pooled sera will need to be equilibrated and validated.

The ability to produce clones of human antibody of a desired subtype to specific antigens provides the opportunity to generate a standardized positive control that is inexhaustible [21]. By using the human combinatorial antibody library, HuCAL, such clones can be identified by combining phage display libraries with recombinant protein expression technology [22]. For recombinant antibody generation, the HuCAL PLATINUM library was used, consisting of a collection of 45 billion fully synthetic human Fab genes with diversified complementarity-determining regions (CDR) inserted into a phagemid vector [23]. Fifteen different antibodies were identified that are specific for Ov16; the two that showed the best binding properties in ELISA and nitrocellulose platforms were selected and converted into human IgG4. One of these recombinant human anti-Ov16 IgG4 clones was used to generate standards for the Ov16 serological ELISA.

Important to understanding the functionality of the positive control antibody in a serological ELISA, two Ov16 ELISA protocols were compared directly to establish a dynamic range and detection limit of the anti-Ov16 antibody for each ELISA method. Since the use of a common control is paramount to data consolidation, a cross-laboratory comparison of ELISA results generated from the positive control antibody was conducted. During optimization of an Ov16 RDT as an alternative immunoassay platform, the positive control antibody was additionally applied for stepwise monitoring of performance through all aspects of manufacturing and use. Because it is not a limited resource or subject to drift in affinity, as may be observed between clinical positive pools, it could be used more widely across QC checkpoints, including during manufacture, storage stability, and during RDT use by surveillance teams. Furthermore, a standardized positive control would support implementation of a QA program to help monitor commercial RDT quality along the delivery chain from manufacturer to end user.

## Methods

### Recombinant antibody selection against Ov16 and conversion into full human IgG4 antibodies

Recombinant antibodies were generated by Bio-Rad AbD Serotec (Puchheim, Germany) from the HuCAL PLATINUM collection of human antibody genes [23] by three rounds of selection on immobilized Ov16 antigen, as previously described [24]. Antibodies in the Fab mini-antibody format Fab-dHLX-FSx2 (bivalent Fab containing a heavy chain C-terminal dHLX-dimerization domain followed by FLAG and Twin-Strep-tag) were screened as crude extracts of E.coli expression cultures in ELISA [25–27]. More than 200 clones were identified which bind Ov16 and do not bind to an unrelated recombinant protein. From sequence analysis of the 25 clones with the highest signal in ELISA, 15 unique antibodies were identified, which were expressed and purified by streptactin affinity chromatography [28]. Specific binding to Ov16 of the purified mini-antibodies was confirmed by ELISA.

Of the 15 clones, two were selected at PATH for conversion into human IgG4 by assessing the binding to Ov16 antigen spotted or striped directly on a nitrocellulose matrix as either a dot blot format or lateral flow format. Binding of the mini-antibodies was detected with the secondary label: peroxidase-conjugated goat anti-human Fab_2_ (Jackson Immuno Research Labs, West Grove, PA). Absence of observable cross-reactivity to GST and WB123-GST was also confirmed on this platform, and two candidate Fab were converted by AbD Serotec to full-length IgG4 constructs using an expression vector system in mammalian cells designed to add the respective constant regions to the light/heavy chain variable components. Screening of the two different IgG4 against Ov16 was performed using Ov16 HRP ELISA (described below) and lateral flow test strips prepared with Ov16 antigen striped onto a nitrocellulose membrane as previously described [16].

The antibody clone AbD19432_hIgG4 was chosen based on both its dynamic range of absorbance values in ELISA and by its greater signal on lateral flow strips, relative to antibody concentration. The concentration of the Ov16 positive control antibody was determined by measuring the absorbance at 280 nm against a 1X PBS blank. The generic immunoglobulin mass extinction coefficient 1.37 (mg/mL)^-1^ cm^-1^ was used to calculate the concentration of the antibody. A 1mg/mL stock of anti-Ov16 recombinant IgG4 in 1X PBS, pH 7.4, is stored at -70°C. When used as a sample in lateral flow tests, the positive control antibody was diluted into either fetal bovine serum, FBS (Thermo Fisher Scientific, Waltham MA) or Ov-negative human serum or plasma and applied in the same manner as a test sample.

### Ethics statement

US-sourced negative sera and plasmas were procured from PlasmaLab International (WA, USA) and Bioreclamation IVT (MD, USA) from donors that had not traveled outside of the United States of America. A clinical Ov-positive specimen pool was made by combining archived plasmas positive for IgG4 antibodies to Ov16 antibody. These archived plasma samples had been collected after written informed consent was obtained from all subjects prior to collection of the samples, and all the subjects consented to having serum or plasma stored for later analysis. All sera or plasmas were stored at -80°C until use. The studies performed in Togo were approved by the PATH research ethics committee and the Togolese Bioethics Committee for Research in Health (Comité Bioethique pour la Recherche en Santé CBRS). Two studies were performed in Togo, one in 2013 (PATH Study File Number HS 716) and one in 2014 (PATH Study File Number 563009–1). All specimens used in this study were collected from study participants who provided informed written consent.

### Ov16 rapid diagnostic test studies in Togo

Two studies were performed in Togo to determine Ov16 seroprevalence by ELISA from DBS and to evaluate early prototype versions of an Ov16 RDT. Both studies were performed during routine onchocerciasis surveillance during the early rainy season prior to MDA: June 4 to July 1, 2013, and May 11 to June 16, 2014. The studies were performed by staff members of the Togo National Program for Onchocerciasis (PNLO) and the Laboratory for Onchocerciasis Research. For each study, the staff received a two-day training course for performing study procedures including appropriate consent, data management, running the RDT, and utilizing the dried-down positive control. In addition, a research team member was present for the first several hundred study participants to monitor test use and to provide ongoing support. Briefly, in the 2013 study, 1,500 subjects were recruited in 15 villages over a period of 27 days, and in the 2014 study, 1,500 subjects were recruited in 20 villages over a period of 38 days.

### Temperature and humidity exposure of RDTs during surveillance activities

The African Program for Onchocerciasis Control (APOC) collected temperature and humidity data during their epidemiological surveillance activities from 2011 to 2012. USB data loggers (Monarch Instruments Inc., Amherst, NH) that record temperature and % relative humidity readings (% RH) were attached to microscopes used by APOC for skin microscopy. The data loggers were set to collect temperature and humidity at a sampling rate of every 15–20 minutes. Since the microscopes were stored at the APOC facilities in Burkina Faso, this dataset allowed collection of transit data as well as operational data. The same temperature and humidity monitors were used during the studies conducted in Togo in 2013 and 2014. In each study, the monitors were similarly attached to the microscopes that were stored and used in parallel to the RDTs.

### Stabilization of monoclonal IgG4 antibody as Ov16 positive control in DBS format for ELISA

Blood samples containing the anti-Ov16 positive control antibody were made by diluting the recombinant IgG4 from a stock solution into FBS (Invitrogen, Grand Island, NY) and then mixing thoroughly at a 1:1 dilution with packed, washed, red blood cells. 75μL per circle marking of the contrived whole blood sample was spotted on Whatman 903 Protein Saver Cards (GE Healthcare, Pittsburgh, PA). These cards were then dried overnight in ambient laboratory conditions, then stored at -20°C with approximately ten cards per sealed foil pouch containing two-unit clay desiccant packets (Desiccare, Reno, NV).

### Alkaline phosphatase (AP)-developed Ov16 ELISA

Immulon 2HB (Thermo Fisher Scientific) plate wells were coated with 100μL of 2μg/mL Ov16 antigen diluted in 0.1M carbonate buffer (Sigma, St. Louis, Mo) overnight at 4°C. DBS were eluted at 2 punches (6 mm circles) in 200μL of PBST + 5% BSA (Sigma) overnight. The following morning, plates were washed four times with PBST (Sigma) and then blocked with PBST + 5% BSA at room temperature. Blocking buffer was removed and DBS eluate samples were added to the plate without dilution at 50μl per well. To prepare dilutions for both the HRP and AP ELISAs, the anti-Ov16 positive control antibody was diluted into PBST + 5% FBS. Samples were incubated at room temperature for two hours, then washed four times with PBST. 50μL of a 1:1,000 dilution in PBST of a biotinylated anti-human IgG4 (clone 6025, Invitrogen) was added to each well. The plates were incubated at room temperature for one hour, and washed four times with PBST. 50μL of a 1:2,000 dilution in PBST of streptavidin conjugated to alkaline phosphatase (Invitrogen) was added to each well. The plates were then incubated at room temperature for one hour, and washed four times with PBST. 50μL of pNPP solution (Invitrogen) was added to each well. Plates were incubated at room temperature for 30 minutes and read at 405nm using an ELISA reader. Replicate well ELISA units were averaged and the limit of detection, LOD, was approximated by determining the first concentration value to produce an average absorbance on or above the mean + 2 standard deviations of the background of the assay.

### Horseradish peroxidase (HRP)-developed Ov16 ELISA protocol

Immulon 2HB (Fisher Scientific) plate wells were coated with 100μL of 5μg/mL Ov16 antigen diluted in PBS, pH 7.4 (Sigma) and left overnight at 4°C. DBS, when used, were eluted at one (6 mm circle) punch in 200μL of PBST+2% milk (Mix’n’Drink, Saco, Middleton, WI) per 6 mm circle punch, overnight. The following morning, plates were blocked with PBST+5%FBS (FBS—Invitrogen) at 37°C. Plates were washed three times with PBST (Sigma) and DBS eluate samples were added neat to the plate at 50μl per well. For plasma or serum samples, a 1:50 dilution was made of each sample in PBST+5%FBS and added to the plate at 50μl per well. The anti-Ov16 IgG positive control antibody was diluted into PBST and 5%FBS. Samples were incubated at 37°C for one hour, then washed three times with PBST. A 1:5,000 dilution of an anti-human IgG4 (6025 clone Hybridoma Reagent Labs, Baltimore, MD) was added at 50μl per well. Plates were incubated at 37°C for one hour, and washed four times with PBST. A 1:10,000 dilution of an HRP-conjugated goat anti-mouse antibody (Jackson Immuno Research Labs) was added at 50μl per well. Plates were incubated at 37°C for one hour, and washed four times with PBST. 100μL of TMB (Sigma) solution was added to each well. Plates were incubated at room temperature for 15 minutes and then the reaction was stopped by adding 50μl per well of 1N HCl (Thermo Fisher). Plates were read at 450nm. Replicate well ELISA units were averaged and the limit of detection, LOD, was approximated by determining the first concentration value to produce an average absorbance on or above the mean + 2 standard deviations of the background of the assay.

### Inter-laboratory use of anti-Ov16 human IgG4 in ELISA

The positive control and ELISA reagents were provided to four independent laboratories: 1) Laboratory of Parasitic Diseases, National Institute of Allergy and Infectious Diseases, National Institutes of Health, Bethesda, Maryland, USA; 2) Global Health Infectious Disease Research Program, Department of Global Health, University of South Florida, Tampa, Florida, USA; 3) Department of Internal Medicine, Infectious Diseases Division, Washington University School of Medicine, St. Louis, Missouri, USA; and 4) Centers for Disease Control and Prevention, Division of Parasitic Diseases and Malaria, Atlanta, Georgia, USA. Specific dilutions of the positive control were run in duplicate wells per plate, over multiple plates, using the same HRP or AP-based ELISA protocols as described above, but with the respective equipment for each lab. Standard curve data submitted from each laboratory for the Ov16 HRP ELISA were compiled for analysis. The coefficient of variation was expressed as the magnitude of the ratio of the standard deviation/mean of all wells’ results at a given concentration across all laboratories.

### Stabilized monoclonal IgG4 antibody as Ov16 positive control for RDTs during surveillance activities

Two formulations of dried Ov16 positive control were prepared. The first was used in an initial laboratory-based pilot study to assess stability and used to accompany an early-stage RDT prototype tested in Togo in 2013 during surveillance activities. For this formulation, recombinant IgG4 was diluted from a stock solution into FBS at a concentration of 2μg/mL. 30μL was added to 1.5mL centrifuge tubes and dried by vacuum centrifugation for 80 minutes at ambient temperature. The tubes were then closed and packaged individually with a ½-gram clay desiccant packet using a heat sealer into pouches in a controlled humidity room with < 20% humidity. To test stability in the laboratory, these pouches were distributed into incubators with the following conditions: 25°C, 45°C, and a variable-temperature incubator with a daily fluctuation between 20°C and 40°C. At specific time points, the dried antibody preparations were rehydrated by adding 60μL of Ov16 RDT running buffer (provided with the Ov16 RDT kit) and 2.5μL was applied to a bare Ov16 lateral flow test strip to assess test-line intensity. The packaged, dried controls were included during RDT testing in Togo in 2013 at 1 control per 50 RDTs. The dried antibody preparation was rehydrated by adding approximately 60μL of Ov16 buffer 20 minutes prior to use.

The second dried formulation was prepared to accompany a late-stage development prototype of a lateral flow-based RDT for Ov16 IgG4 (Ov16 RDT), tested in Togo during MDA and surveillance activities in 2014. Recombinant IgG4 was diluted from a stock solution into FBS at a concentration of 250ng/mL. 30μL per tube was added to 1.5mL centrifuge tubes and left open at room temperature overnight, leaving a film coating the walls of the tube. The tubes were then packaged in the same manner as the first formulation. One control pouch was incorporated with each kit of 25 RDTs. The team was instructed to run a positive control every time a box of 25 RDTs was opened to verify the kit was functional. The positive control was rehydrated by adding four drops, approximately 75μL, of Ov16 buffer, allowing the pellet to dissolve for 20 minutes, and then running the rehydrated control solution using the same protocol as a test sample. To run test blood or plasma samples, 10μL of sample was collected in the sample transfer pipette. The sample was added to the sample port by gently squeezing the pipette to release the sample into the port. Four drops (approximately 75μL) of Ov16 buffer was added to the buffer port. The test was read at 20 minutes.

## Results

### Recombinant antibody selection

**Fig 1:**
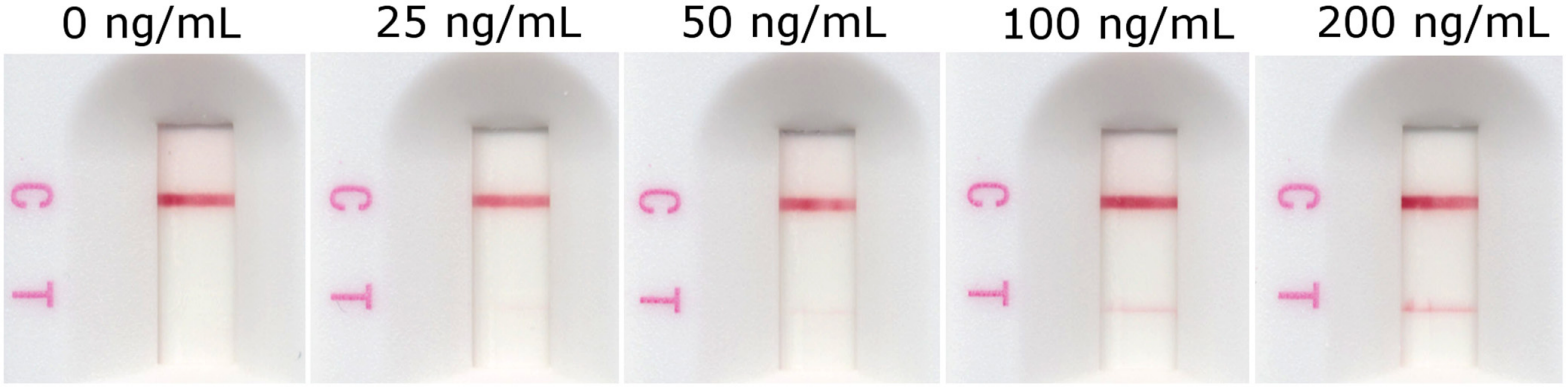
Dose-dependency of the intensity of the RDT (SD BIOLINE Onchocerciasis IgG4) test line result to increasing concentrations of the anti-Ov16 human IgG4 spiked into pooled normal human plasma. Ten microliters of the plasma is applied as sample and run according to manufacturer’s instructions.

**Fig 2:**
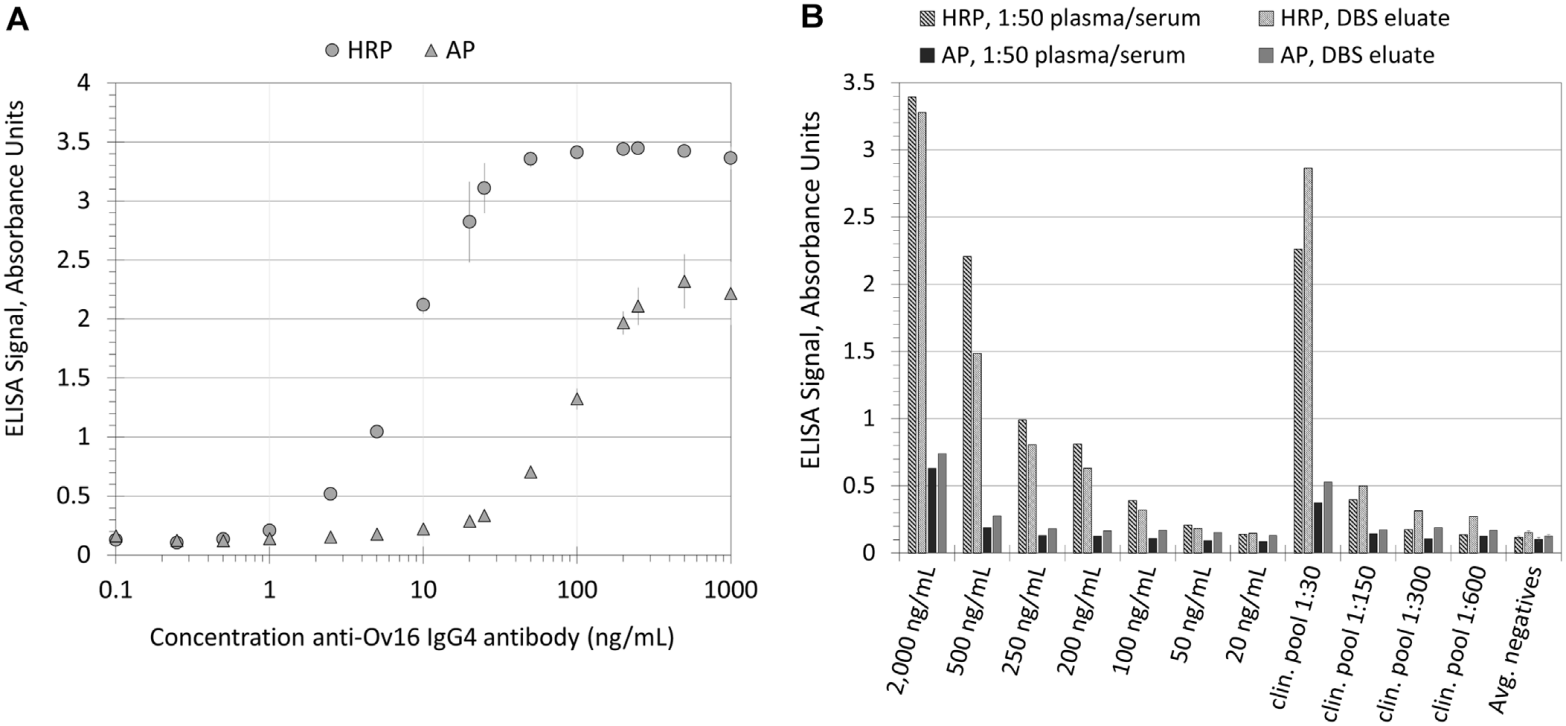
Concentration-dependent absorbance values of the ELISA platform for the anti-Ov16 human IgG4 serological assay. (A) Absorbance units versus concentration of recombinant anti-Ov16 IgG4-positive control antibody. (B) HRP ELISA and AP ELISA absorbance values from both plasma/serum and DBS samples derived from identical sources: anti-Ov16 IgG4-positive control-spiked negative human sera, dilutions of an Ov-positive pool from microfilaria-positive patient plasmas and sera, and the average of nine negative plasmas and sera (from donors that have not traveled to an Ov-endemic area).

The Fab phage display library HuCAL PLATINUM was used for the generation of antibodies binding to recombinant *O*. *volvulus* antigen Ov16. Fifteen antibodies in bivalent Fab mini-antibody format were selected and tested in spot and lateral flow assay as well as in ELISA. Two clones, AbD19432 and AbD19422, were selected for conversion to full human IgG4 based on performance in the spot and lateral flow test (signal-to-noise ratio in serial dilutions). While both clones produced soluble IgG4 with similar performance on the Ov16 ELISA and lateral flow strips, clone AbD19432_hIgG4 was selected as the final candidate for the study primarily based on a slightly more intense result than the other clone at lower concentrations when used with the RDT. Dose-dependent results were observed both on RDTs and in ELISA (Figs 1 and 2). A 10 μL sample of 25 ng/mL run on the RDT is visible by eye as a very weak positive test line.

### Performance of Ov16 positive controls on ELISA for anti-Ov16 human IgG4

Dilutions of the Ov16 positive control were assayed using both the HRP Ov16 ELISA and the AP Ov16 ELISA. Fig 2A shows dose dependence of ELISA signal for both ELISA methods. The analytical limits of detection for the HRP and AP ELISA were approximately 1ng/mL and 10ng/mL antibody, respectively. Fig 2B shows comparison of the HRP and AP ELISA results for positive control-spiked sera and clinical positive pool sera prepared as DBS and eluted with the same samples processed as plasma/serum samples (1:50 dilution). The DBS specimen type requires the input of approximately 9 μL of plasma or serum per 6mm punch per ELISA evaluation as compared to only 2 μL for plasma serum when diluted 1:50. However, similar OD were obtained for paired specimen types by ELISA for both HRP and AP ELISA methods. Differences between the ELISA methods were similar to results obtained with the dilution series of positive control; the HRP ELISA had a lower limit of detection than the AP ELISA as well as a broader dynamic range (Fig 2B) and the HRP DBS ELISA protocol required only 1 punch instead of the 2 punches used in the AP ELISA protocol. Additionally, true positive patient pools were found to have a higher signal relative to negative sera when run with the HRP ELISA, suggesting the capacity for higher signal-to-noise ratios.

### Inter-laboratory comparison of HRP ELISA

**Fig 3:**
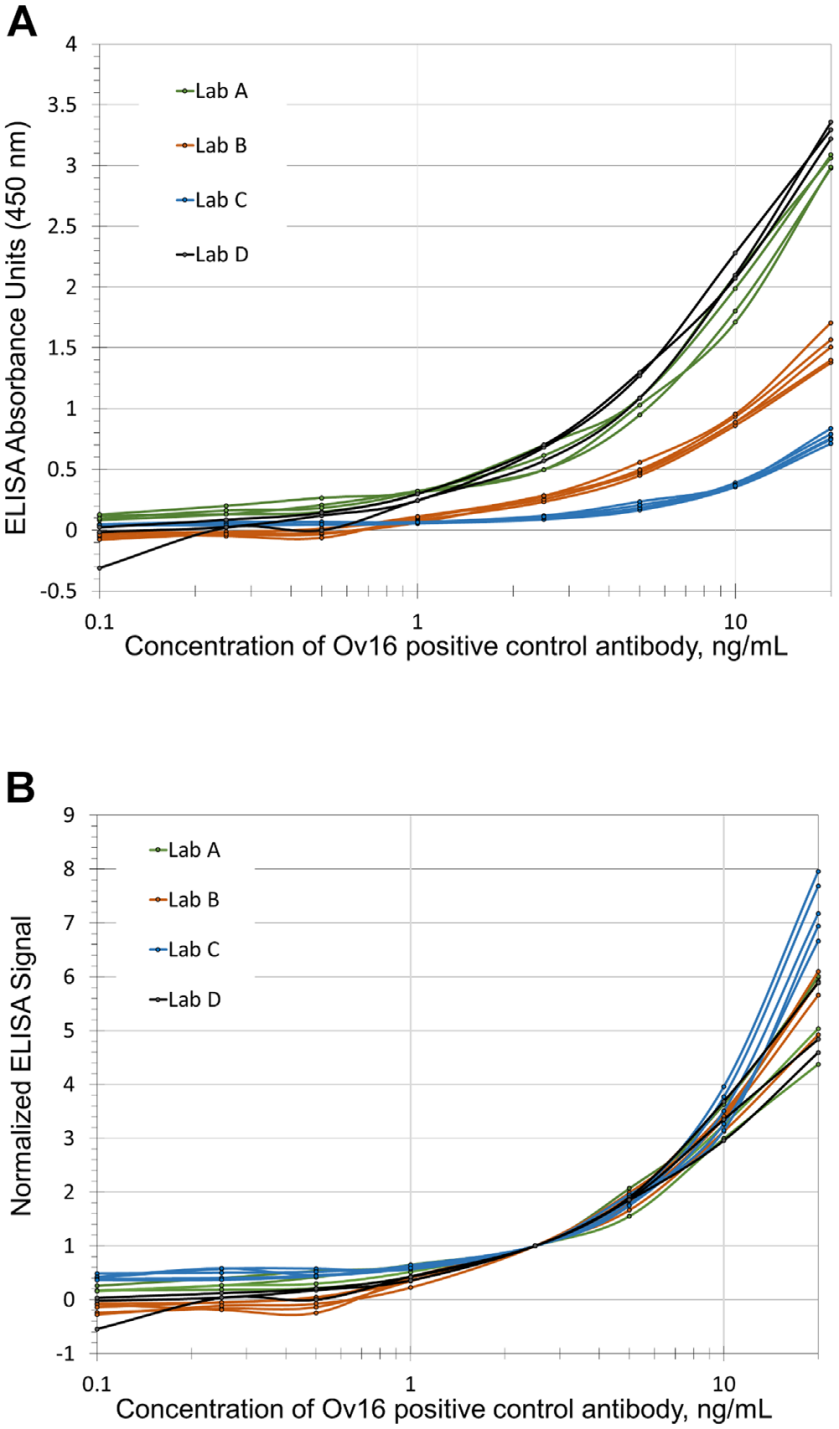
(A) Combined inter-laboratory HRP ELISA signal profiles for anti-Ov16 human IgG4 antibody. Concentrations between 0.1ng/mL and 20ng/mL were assayed in replicate ELISA plates by four different laboratories using the same protocol and starting reagents. Each plate contained the concentration range run in duplicate wells. Different laboratories, identified as A-D, ran between three and five replicate plates. Shown is data collected from the laboratories (color-grouped) with each line representing the profile from a single plate of the averaged duplicate wells for each concentration tested. (B) Processed data representing the same data with the same color-coding as shown in 3A, but shown with absorbance values normalized to each plate’s respective optical absorbance at 2.5 ng/mL of control antibody.

Fig 3 shows comparison of standard curves of positive control dilutions, run using the HRP ELISA method, from the participating laboratories. The raw or plate background-subtracted data showed a diverse range of absorbance values with respect to concentration of the positive control, highlighting inter-laboratory variability (Fig 3A). As demonstration that the anti-Ov16 IgG4 antibody can be used as a positive control, the plate absorbance values and standard curves were then normalized to the average value of the 2.5ng/mL sample from their respective plates. The resulting normalized data show relatively cohesive absorbance values, particularly in the linear range of the assay (Fig 3B). While the curves disperse significantly at extreme ends of the absorbance range, the normalization permits comparison between multiple plate datasets. For ELISA results produced by 1ng/mL positive control (near detection limit), the magnitude of the coefficient of variation (CV) decreased from 0.67, for all points from all laboratories prior to normalization to 0.24, following normalization. For a given concentration, normalization did not always produce a reduction in the magnitude of the CV using the single-point normalization method. However, the average of all inter-laboratory CV values for given concentration points went from 2.29 before normalization down to 0.79 after normalization.

### Environmental conditions for transit and operation of diagnostics during onchocerciasis surveillance activities

Positive controls for RDTs and the RDTs themselves were exposed for prolonged periods of time to a broad range of temperature and humidity conditions during surveillance activities. In order to capture the range of these conditions, temperature and humidity measurements were collected from a series of APOC surveillance activities and two surveillance activities in Togo.

Temperature and humidity monitors were attached to each of two microscopes used for skin snip microscopy, sent from Burkina Faso to five APOC epidemiological surveillance sites in 2011 and 2012. These included the foci: Logon Occidental, Logon Oriental, and Moyen-Chari in Chad June to August 2012; Kilosa in Tanzania April 2012; Enugu in Nigeria September to November 2011; Adjumain and Moyo in Uganda July to August 2012; and Kasese in Uganda July to August 2012. The studies included temperature and relative humidity data collected during transit and the period throughout which the surveillance was carried out. The summary statistics are shown in Table 1. The largest temperature and relative humidity variation was observed during shipment of the microscopes, 8.2°C –37.9°C and 40.2% to 119.5%. The infrequent values >100% relative humidity were possibly due to artifacts from brief temperature fluctuations which could affect the relative humidity reported, or possibly due to moisture collection on the monitors. Such values would be interpreted as near 100% humidity. Most critically, the tests and positive controls needed to demonstrate stability over a temperature range of 17.6°C–37.8°C and a relative humidity of 40.2% to 119.5% as observed during performance of surveillance activities.

**Table 1:** Summary statistics for temperature and humidity exposure experienced by microscopes used by APOC during (1) transit to surveillance sites and (2) during epidemiological surveys in the years 2011 and 2012. (3) The summary statistics for the operational temperature experienced by Ov16 RDTs during 2013 and 2014 surveillance activities in Togo.

	Maximum	Minimum	Mean	Median	Standard Deviation	Data Points
**(1) Temperature and humidity statistics during transit to APOC surveillance site**						
Temperature	37.9°C	8.2°C	25.1°C	25.1°C	3.5°C	7,740
Relative humidity	116.5%	18%	69%	73%	20.3%	7,740
**(2) Temperature and humidity statistics during APOC surveillance activities**						
Temperature	37.8°C	17.6°C	26.6°C	26.5°C	2.8°C	18,000
Relative hucmidity	119.5%	40.2%	70.4%	72.1%	10.9%	18,000
**(3) Temperature and humidity statistics during Togo 2013 and 2014 studies**						
Temperature	40.1°C	9.0°C	28.7°C	28.4°C	3.0°C	20,286
Relative humidity	96.5%	32.9%	66.4%	67.6%	8.0%	20,286

**Fig 4:**
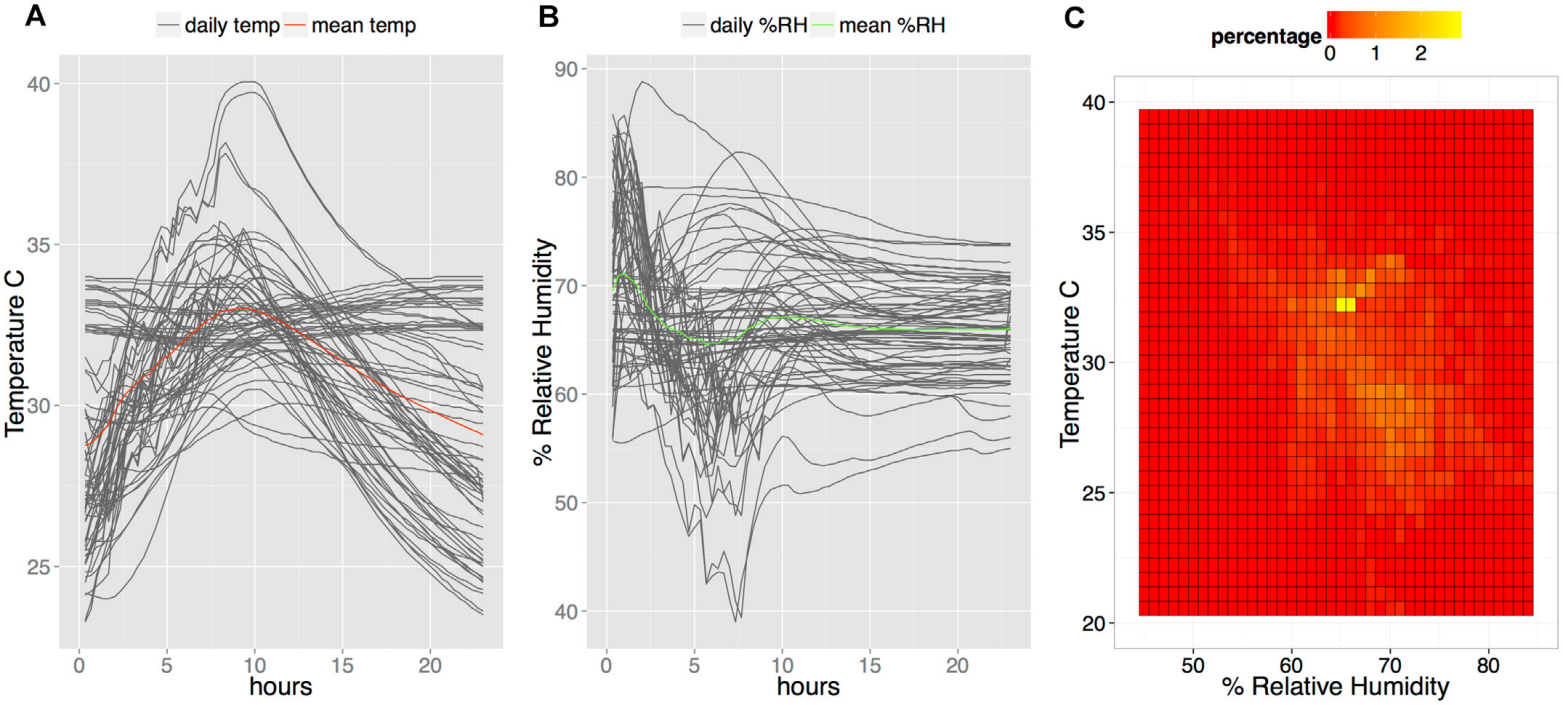
Environmental conditions during surveillance activities in Togo 2013 and 2014. A. Daily temperature profiles (dark grey lines) in degrees Celsius during surveillance activities. The mean temperature is plotted with the bold red line. B. Daily % relative humidity (dark grey lines) cycles during surveillance activities. The mean relative humidity is plotted with the bold green line. C. Frequency of recorded temperature and relative humidity pairs experienced by monitors attached to microscopes used during the surveillance activities. The color scale represents the frequency of observations represented by intense red (least frequent) to intense yellow (most frequent).

Temperature and relative humidity data were also collected during two Togo MDA and surveillance activities in 2013 and 2014 in which the positive control was used to verify functionality of every new RDT kit used as soon as the kit was opened (Table 1 and Fig 4). This data represents the operational temperature and relative humidity conditions to which the packaged tests and positive controls were exposed during these activities.

### Dried positive control for use with RDTs

**Fig 5:**
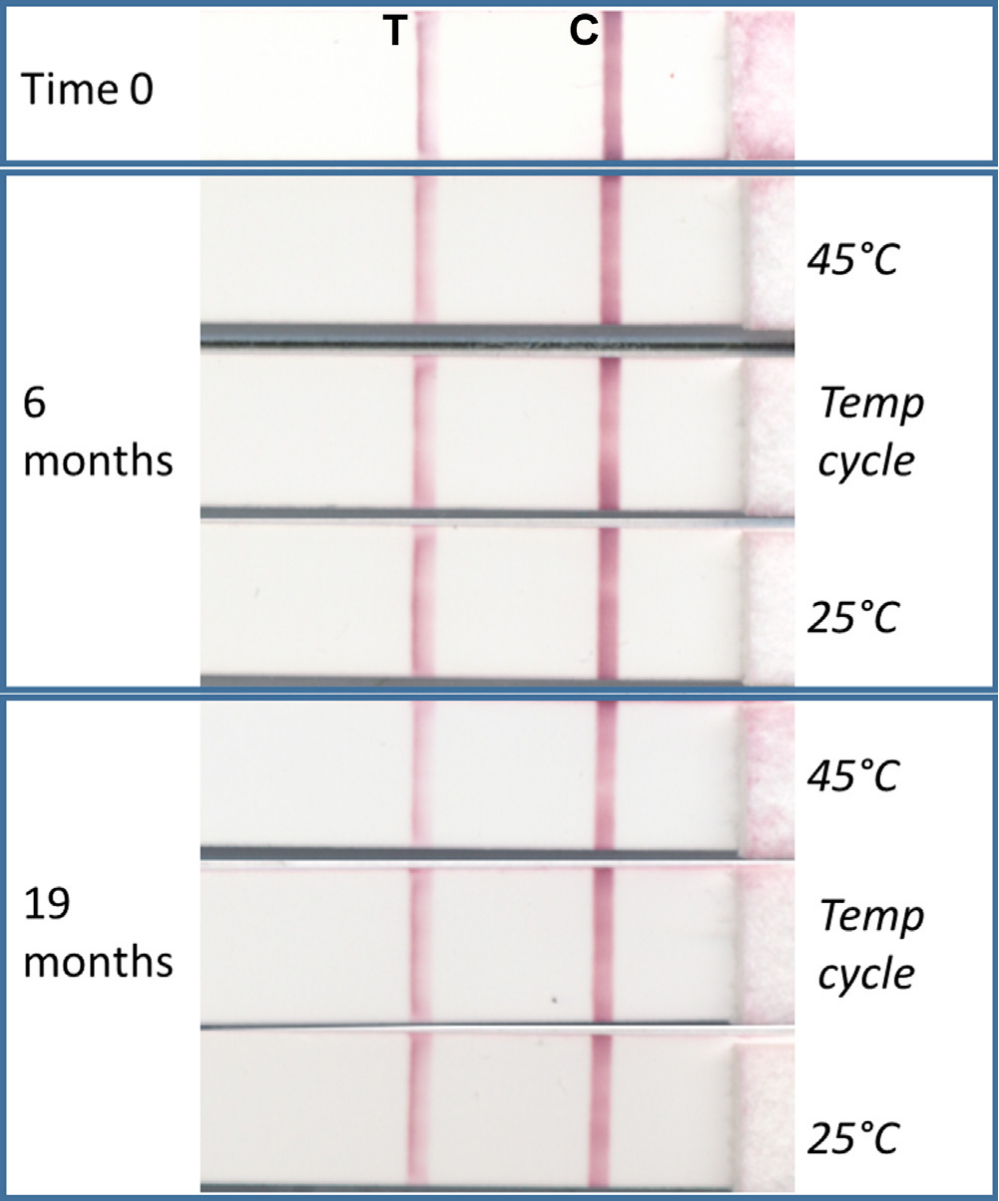
Lateral flow test strip scanned images showing test line (‘T’, left) and control line (‘C’, right) results after using as samples the dried anti-Ov16 IgG4-positive control subjected to elevated temperature stress over time. Time zero shown indicates intensity of the starting signal prior to storage at 25°C, 45°C, and variable cycling temperature (20°C to 40°C, daily) for up to 19 months.

**Fig 6:**
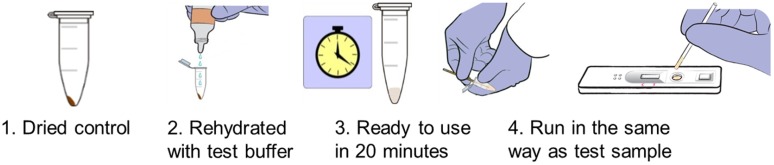
Workflow for use of dried positive control with the Ov16 RDT.

Generating a dried form of recombinant IgG4 creates an opportunity for a stable antibody available for use in the absence of colder temperature storage. The first formulation was tested for stability in the laboratory using high temperature and daily temperature cycling conditions and was found to produce a clear positive signal after over 1.5 years of high-temperature stress (Fig 5). Despite indication of stability in that all positive controls gave a positive signal by RDT throughout the 2013 study, the vacuum-drying method produced a friable pellet which could break and fragment throughout the tube during transport, as noted during the study. An alternative method of drying-down the positive control, compatible with transport and with the SD BIOLINE Ov16 RDTs, was used to produce a thin film at the bottom of the tube which was less likely to fragment. This type was used in the study conducted in 2014. Both types of dried positive control were designed so that the user need only add 2 or 4 drops of Ov16 buffer (depending on dropper bottle type used in study) to the vial, rehydrate the positive control for 20 minutes, and then run the Ov16 RDT as per a normal sample (Fig 6). Observation during training of the PNLO surveillance team using the dried-down positive control in the field indicated that this process was simple and easy to perform. This second formulation was used to monitor the performance of RDTs throughout the duration of a surveillance and MDA activity in Togo in 2014. In brief, 60 kits of RDTs were used over a period of 38 days (May 11, 2014, to June 16, 2014) during which time both the RDTs and the dried positive control, packaged as individual packets similarly to an RDT, were exposed to the environmental conditions shown in Fig 4. All 60 positive controls gave a positive signal, showing a minimum operational stability at ambient and operational environmental conditions of 90 days (starting from date of production of the dehydrated positive controls through final use during field study).

## Discussion

The human IgG4 antibody response to *O*. *volvulus* antigen Ov16 has been identified as a specific serologic marker of exposure to *Onchocerca volvulus*[11] and has been accepted to monitor progress toward elimination of river blindness [29]. To address a need for a consistent, standardized, and widely accessible source of positive control, a recombinant human IgG4 with specific affinity to the Ov16 antigen was generated.

Evaluation of the anti-Ov16 IgG4 antibody by ELISA demonstrated a significantly better limit of detection, and favorable signal-to-noise ratios when used with the HRP ELISA method as compared to the AP ELISA method. Differences between the outcomes might be explained by variation of the components in the two different ELISA protocols, such as blocking buffer or antigen-coating concentration and buffer. When such variables were tested as individual changes to the protocol, results did not explain the observed difference in detection limit. Since the AP ELISA uses the same clone of anti-human IgG4 as the HRP ELISA protocol, antibody affinity alone is also insufficient to explain the difference. While data presented here implies the HRP ELISA as having more favorable analytical performance, more studies with clinical specimens would be required to understand its utility for surveillance purposes, and in particular, the relative specificities of the assays. The AP ELISA has already been used in multiple countries as a tool to verify transmission interruption and elimination [4,6,7,14,15]. In an elimination context, high specificity of an assay, along with high quality control and reproducibility of the assay, is essential to minimize follow-up on false positives.

The positive control described in this study can be used with either ELISA method as either plasma or DBS sample type. This comparison allowed sample type comparison, ELISA type comparison, and showed the feasibility of the positive control for use in multiple specimen formats. Use of such a monoclonal antibody to generate data regarding the relationship between OD of positive control and the assay’s sensitivity and specificity towards clinical samples should aid in ongoing data comparison to help answer research questions that are key to understanding transmission indicators such as antibody persistence and intensity of antigen-specific antibody responses from individual in regions with varying prevalences and stages of elimination of *Onchocerca volvulus*.

In addition to standard curve dilutions or controls for ELISA, the positive control can be used to produce a relevant process control for assays sourcing specimens from DBS. Implemented in ELISAs run in Sokodé, Togo, a DBS control prepared in the lab with a serum concentration of positive control of 250 ng/mL was used in the HRP ELISA as a process and normalization control for each ELISA plate. From a cost perspective, approximately US$1 of positive control antibody can be used to make several milliliters of spiked positive control serum supplying over 200 ELISA assays with standardized positive control in this dried blood spot (DBS) form. Prepared more simply as a solution of 2.5 ng/mL to use as a plate control, the same amount can supply hundreds of thousands of ELISA plates. As demonstrated from the comparison of ELISAs performed across four laboratories, the anti-Ov16 IgG4 control may facilitate ELISA plate QC and data normalization for data analysis and compilation. A positive value in the linear absorbance range may be less influenced by assay limits and a simple control requiring little handling will be less prone to variation. These parameters should guide choice of form and concentration for normalization. A positive control should be run with every plate, making access to a universal positive all the more critical. Understanding the absorbance result of a given concentration of positive control that optimizes the definition of true positive and true negative from endemic regions requires continued data collection.

With the availability of an Ov16 RDT (SD BIOLINE Onchocerciasis IgG4 test; Alere, Standard Diagnostics, Inc., South Korea), the ability to monitor the quality of an RDT outside the context of a laboratory throughout the lifecycle of the test in shipping, storage, and use is paramount to having reliable results. Point-of-care assays and tools used in surveillance applications must withstand demanding environmental conditions in order to be appropriate and impactful for even remote populations in need, as demonstrated from the data collected for this study across seven onchocerciasis surveillance activities between 2011 and 2014. Having a portable, stable, positive control that can withstand the same conditions is a key component of this quality monitoring. The simple dry formula, designed for use with the RDT running buffer, demonstrated feasibility as a QA reagent, robust enough as packaged to withstand the stress of temperature and humidity variation without the requirement of cold chain in surveillance activities performed in Togo. This, combined with laboratory stability data that shows little decline in antibody affinity after 19 months at 45°C, suggests that a dried formulation of the recombinant IgG4 antibody is robust enough to be a practical option as an accessible positive control reagent. While in these studies the positive control was run using the Ov16 RDT at a high rate of 1 per 25 tests in order to monitor both the RDT and the positive control itself, ideally the positive control antibody will be used in a mature quality assurance program that will provide recommendations on sampling rates and specific concentrations needed.

Use of a single positive control can provide a performance link between ELISA and rapid diagnostic tools, such as the SD BIOLINE Onchocerciasis IgG4 test, to understand limits of detections of clinically-relevant antibody responses. Currently, 10μL of 25 ng/mL positive control in plasma can be detected on the RDT as a weak positive. If such concentration were a test sample, it would by process be diluted 50-fold for use in ELISA. This would produce a final concentration of 0.5 ng/mL, which as compared to typical standard curve responses, would barely be detected above baseline. This can be seen in Fig 2B when concentrations of positive control are run as test samples. In this case, The RDT and the HRP ELISA procedure results in similar detection limits of a given concentration. However, caution should be used when making direct comparisons between LOD across the ELISA platform and RDT platform since the LOD of a monoclonal antibody can be assay-specific while important measures of performance such as sensitivity and specificity can only be determined using real clinical samples. Determining the relationship between performance with clinical samples and positive control results must be made for each assay type before performance conclusions can be drawn based on the LOD of the positive control.

The approach described in this study should be generalizable to other similar serology-based markers for exposure to pathogens, such as IgG4 specific to the lymphatic filariasis-associated *Brugia malayi* and *Wuchereria bancrofti* markers BM14 and WB123, respectively. Serology marker-based assays represent an opportunity to integrate surveillance activities for multiple pathogens [30,31]. Positive control reagents that can function cross-platform as broad-spectrum QA reagents will help ensure these assays can play the intended role in surveillance for transmission of these pathogens. While a monoclonal antibody control minimizes the risk of lot-to-lot heterogeneity and potential drift of clinical positive pools, it cannot be used as a substitute to clinical panels to measure assay performance and caution should be used if applying the positive control antibody to compare performance cross-platform given potential differences in epitope availability between assays. For example, a change in immunoassay platform may greatly affect detection limits of the control antibody but have little effect on clinical detection limits. When used as a QC reagent for the Ov16 antigen itself, the limitations and advantages of a monoclonal antibody should be considered. Limitations may include lack of detection of antigen mutation outside the epitope of the Ov16 protein. Advantages may be the positive control’s sensitivity to epitopic mutation in the antigen and less drift in the affinity of the control itself. Future work may include epitope mapping and comparison of positive control and positive pool samples with different lots of antigen to establish baseline lot-to-lot variability. For quality control, the parameters for positive control use should be assay-specific and well characterized prior to implementation.

A comprehensive QC and QA program ensures that a diagnostic test will provide the desired output from its use—a consistent and reliable detection of analyte. QC is a product-based approach implemented at the manufacturing level that is reactive or corrective to identified defects. QA is a process-based approach that comprises procedures and systems to ensure all deliverables are consistently of good quality. Production of the anti-Ov16 antibody-positive control can support QC and QA programs for Ov16-based onchocerciasis diagnostics through incorporation into training aids, QA panels, and reagents to support optimal test use and standard operating procedures (SOPs) for ELISA standardization.
